# Medical management of pleural infection: Why not saline intrapleural lavage?

**DOI:** 10.1111/crj.13548

**Published:** 2022-09-29

**Authors:** Valentine Mismetti, Marios E. Froudarakis

**Affiliations:** ^1^ Department of Pulmonology and Thoracic Oncology, North Hospital University Hospital of Saint‐Etienne Saint‐Priest‐en‐Jarez France

In the last two decades, significant effort has been performed to understand and manage pleural infections. However, pleural infection remains a major health issue associated with long hospitalization, high morbidity, and mortality rates. Indeed, pleural infection leads to up to 21 days of hospital stay, about 30% of patients will undergo a thoracic surgery procedure for their pleural infection, and mortality rates vary between 10% and 20% according to age.^1^, ^2^ The goal of pleural infection management is the prompt treatment of sepsis^3^ in order to improve morbidity and mortality, avoid surgical treatment, and reduce the length of hospitalization and total costs.^4^ The presence of loculations and pus are significant factors related to the treatment failure.^5^ Patient's rapid diagnosis and monitoring are important, as clinical presentation varies and the use of different radiological and laboratory means plays an important role in the patients' management.^6^

The cornerstone of the therapeutic management remains antibiotic therapy and pleural drainage.^7^, ^8^ Antibiotics are generally initiated intravenously and empirically because up to 40% of cases will remain negative in cultures.^1^ Community‐acquired pleural infections should target gram‐positive aerobes, as well as anaerobes, as these organisms have a much lower positive culture yield. For nosocomial infections, antibiotics should cover methicillin‐resistant *staphylococcus aureus* and anaerobes.^9^ Pleural penetrance has been demonstrated in most classes of antibiotics, with the exception of aminoglycosides.^7^ The duration of antibiotic therapy in pleural infection has not been assessed in detailed clinical trials; however, antibiotics are often continued for at least 3 weeks and the switch from intravenous to oral being guided by clinical, biological, and radiological improvement.^7^

Pleural drainage is indicated in the presence of pus or positive pleural fluid culture, loculations on chest ultrasound (or computed tomography), or an effusion covering at least 50% of the hemithorax.^4^ A pleural fluid pH of <7.20 also indicates drainage, under the condition that it has been taken and analyzed correctly and bearing in mind that different loculations may have different pH.^10^ The size of the chest tube is the subject of an ongoing debate; however, it was suggested that chest drains with smaller diameters cause less pain, with no difference in clinical outcome for the treatment of pleural infection.^11^ Intrapleural fibrinolytics have been tested in small phase II clinical trials in the late 90s and early 2000s with promising results.^3^ However, MIST‐1 trial comparing streptokinase to placebo with the aim to reduce mortality (primary outcome), hospital stay, and surgical referral (secondary outcomes) showed no difference between the two groups.^12^ The results of MIST‐1 were debated as major criticisms have been raised, including that the patients before being enrolled in this trial had a significant period of observation.^3^ The following MIST‐2 trial showed significant effect in improving chest X‐ray (primary outcome), although failed to enroll the predicted number of patients, reducing the surgical referral and the length of hospital stay with the combination of r‐TPA to DNAse.^13^ However, the dosing of r‐TPA/DNAse was empirically chosen, while alteplase may be the cause of severe intrapleural bleeding,^14^ and, therefore, recent articles stepped down to r‐TPA 5 mg with equal benefit, less side effects, and lower cost.^15^ A controlled trial^16^ comparing urokinase 100 000 UI to alteplase 10 mg enrolling 99 patients showed equal effect on success rates, surgical referral, or mortality, while bleeding was significantly higher with rTPA. The recent Cochrane meta‐analysis^17^ comparing intrapleural fibrinolytic therapy to placebo concluded that fibrinolytics were associated with a reduction of surgical intervention and overall treatment failure but with no evidence of change in mortality. Concerns were raised for the high rate of bleeding with rTPA.^17^

Another option for intrapleural therapy may be pleural irrigation with normal saline. The idea behind is to dilute and remove bacteria, cytokines, inflammatory cells, and pro‐fibrinogenic coagulation factors, which induce pleural fluid organization. Also, the mechanical process of irrigation increases pleural fluid drainage by reducing stasis and organization of the intrapleural contents.^18^ To this end, the pleural irrigation trial (PIT)^18^ enrolling 35 patients showed promising results using 250 ml of 0.9% sodium chloride delivered by an infusion set into the pleural cavity 3 times per day, for 3 days. Thus, saline irrigation resulted in a 32.3% reduction in computed tomography‐assessed pleural collection volume, compared to 15.3% receiving standard care (*p* = 0.03) and referral to surgery (*p* = 0.03), with no difference in mortality rates.

In this issue of “The Clinical Respiratory Journal,” Guinde et al.^19^ report results from a retrospective analysis of 30 patients (11 of them having active neoplasia) undergoing normal saline pleural lavage for pleural infection. The primary outcome was the rate of failure at 3 months. Only four patients (13.3%) failed initial treatment at 3 months.^19^ No patient was referred to surgery; the median length of hospital stay was 17 days (11–42) for a quite long median pleural drainage of 14 days (7–28). Additional pleural procedures were reported in three patients (10%) with no other complications, and the only death reported was a patient with lung cancer in palliative care.^19^ Despite limitations of this study related to the lack of specific therapeutic protocol due to its retrospective nature, the small sample size, and the absence of control group, the results of Guinde and collaborators are interesting specifically because they concern frail patients. Saline intrapleural lavage (SIL) appears to be an effective and safe procedure for the medical management of pleural infection, yet this data need to be further confirmed by large, controlled studies.

## CONFLICT OF INTEREST

Authors state no conflict to disclose or funding source.

## AUTHOR CONTRIBUTIONS

Both authors have equally contributed to the manuscript.

## Data Availability

Data sharing not applicable to this article as no datasets were generated or analysed during the current study.
